# ChemDIS-Mixture: an online tool for analyzing potential interaction effects of chemical mixtures

**DOI:** 10.1038/s41598-018-28361-6

**Published:** 2018-07-03

**Authors:** Chun-Wei Tung, Chia-Chi Wang, Shan-Shan Wang, Pinpin Lin

**Affiliations:** 1 School of Pharmacy, Kaohsiung Medical University, Kaohsiung, 80708 Taiwan; 2 Department of Medical Research, Kaohsiung Medical University Hospital, Kaohsiung, 80708 Taiwan; 3 National Institute of Environmental Health Sciences, National Health Research Institutes, Miaoli County, 35053 Taiwan; 4 Research Center for Environmental Medicine, Kaohsiung Medical University, Kaohsiung, 80708 Taiwan

## Abstract

The assessment of bioactivity and toxicity for mixtures remains a challenging work. Although several computational models have been developed to accelerate the evaluation of chemical-chemical interaction, a specific biological endpoint should be defined before applying the models that usually relies on clinical and experimental data. The development of computational methods is desirable for identifying potential biological endpoints of mixture interactions. To facilitate the identification of potential effects of mixture interactions, a novel online system named ChemDIS-Mixture is proposed to analyze the shared target proteins, and common enriched functions, pathways, and diseases affected by multiple chemicals. Venn diagram tools have been implemented for easy analysis and visualization of interaction targets and effects. Case studies have been provided to demonstrate the capability of ChemDIS-Mixture for identifying potential effects of mixture interactions in clinical studies. ChemDIS-Mixture provides useful functions for the identification of potential effects of coexposure to multiple chemicals. ChemDIS-Mixture is freely accessible at http://cwtung.kmu.edu.tw/chemdis/mixture.

## Introduction

Human beings are constantly exposed to mixtures of multiple chemicals. Simultaneous exposure to multiple chemicals could lead to complicated results compared with the exposure to individual chemicals^1^. While many prediction methods have been developed for potential effects associated with a single chemical, little progress has been made toward the computational identification of potential interaction effects between multiple chemicals. The development of computational methods for identifying the most potential effects of coexposure to multiple chemicals from numerous biological endpoints is highly desirable.

In contrast to direct chemical-chemical interactions which could be predicted by using chemical structure information^2^, the identification of potential indirect chemical-chemical interactions which disturb common targets or pathways remains a challenge. Several methods are proposed to predict the outcome of indirect chemical-chemical interactions based on individual experimental results. For example, concentration addition and independent action models are respectively applied to mixtures with shared targets and independent mode-of-action^3,4^. Biomolecular interaction networks have also shown potential for prediction and analysis of synergistic effects of drug combinations^5–10^. However, the abovementioned methods are only applicable to chemicals with a known common endpoint. There is a strong unmet need for the early identification of potential endpoints including target genes, pathways, functions and diseases.

Chemogenomics-based systems such as ChemDIS^11^ and Comparative Toxicogenomics Database (CTD)^12^ have been established to support the inference of affected functions, pathways and diseases associated with a single chemical using chemical-gene/protein interaction profiles. The development of computational tools for integrative analysis of chemogenomics data from multiple chemicals could be useful for identifying potential endpoints of coexposure to multiple chemicals.

Our present study presents a novel tool named ChemDIS-Mixture for the analysis of potential coexposure effects, based on our previous ChemDIS system that has been successfully applied to the disease inference for various studies^13–15^. The shared interacting gene targets and enriched functions, pathways and diseases will be automatically identified with a joint *p*-value for prioritizing the potential interaction effects of coexposure. In addition, the enriched analysis of functions, pathways and diseases for all interacting genes will be calculated representing the overall effect of the coexposure. The analysis functions of ChemDIS-Mixture were demonstrated by two case studies.

## Results

**Figure 1:**
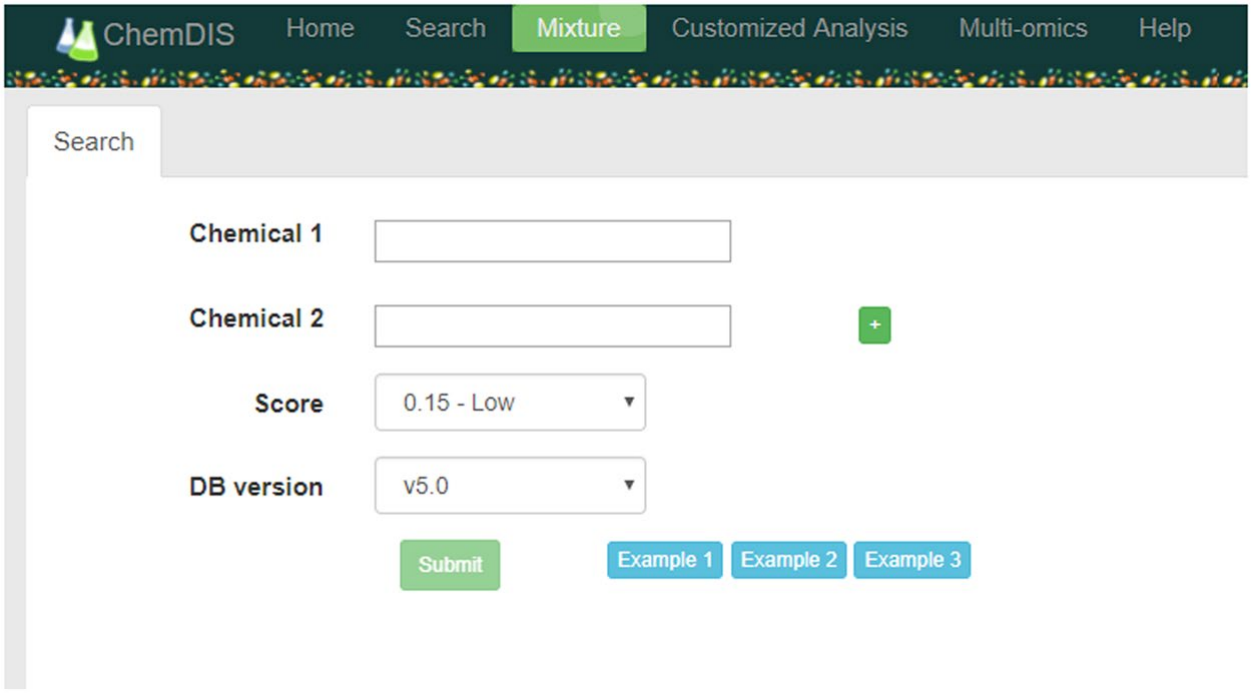
The user interface of ChemDIS-Mixture. The plus icon is clickable for appending up to four chemicals for analysis. The score for filtering out low-confident chemical-protein interactions can be specified. The newest database version (default) is recommended.

**Figure 2:**
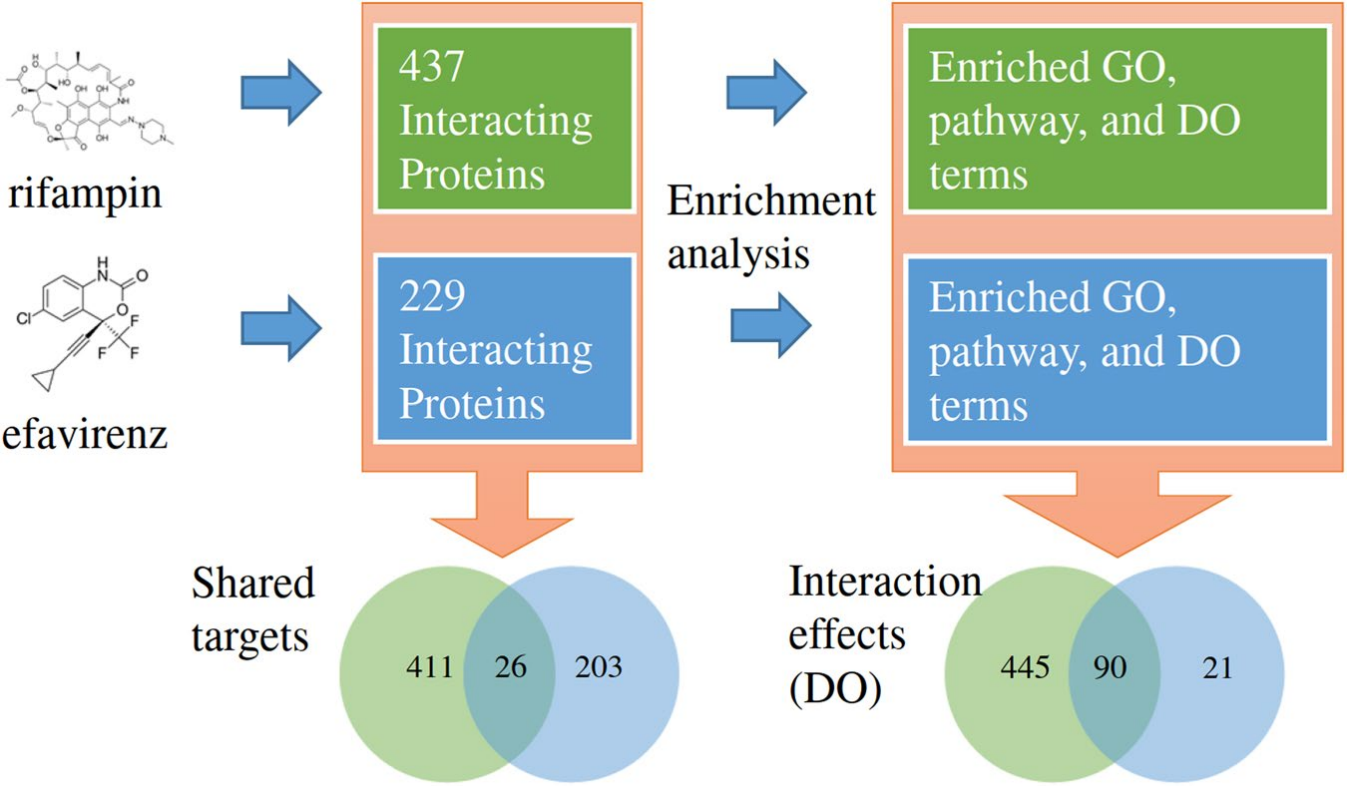
An illustrative flowchart of ChemDIS-Mixture for two chemicals.

We have developed a novel tool ChemDIS-Mixture for the analysis of chemical-chemical interactions with associated interacting protein data. The utilization of STITCH database^16^, the largest chemical-protein interaction database integrating several databases such as ChEMBL^17^, CTD^12^ and DrugBank^18^, enables the analysis for more than 430,000 chemicals. Currently, only human chemical-protein interaction data are integrated into ChemDIS-Mixture. The user interface for ChemDIS-Mixture is shown in Fig. 1. Autocomplete function has been implemented to help the selection of chemicals with available chemical-protein interaction data. CAS numbers are also acceptable for querying chemicals. Currently, up to four chemicals can be simultaneously analyzed using ChemDIS-Mixture for the sake of intuition. We are working on an extended version dealing with more chemicals that will soon be available. Users can specify the score threshold for filtering interacting proteins based on its confidence. Three levels of scores for identifying low, medium and high confidence interacting proteins for subsequent analysis have been defined according to STITCH database^16^. While STITCH database versions of 4 and 5 can be specified, the latest version providing more comprehensive data is recommended. An illustrative flowchart is shown in Fig. 2. For each input chemical, its interacting proteins will be extracted and enrichment analysis will be conducted based on a hypergeometric test for identifying the enriched GO, pathway, DO and DOLite terms with an adjusted p-value < 0.05 using Benjamini-Hochberg multiple test correction^19^.

For each analyzed chemical, basic structure and property information including the chemical 2D structure, hydrogen-bond acceptor, hydrogen-bond donor, IUPAC name, InChI, InChIKey, molecular formula, molecular weight, canonical SMILES, isomeric SMILES and topological polar surface area (TPSA) is available at ChemDIS-Mixture with hyperlinks to PubChem, 2D and 3D structures.

**Figure 3:**
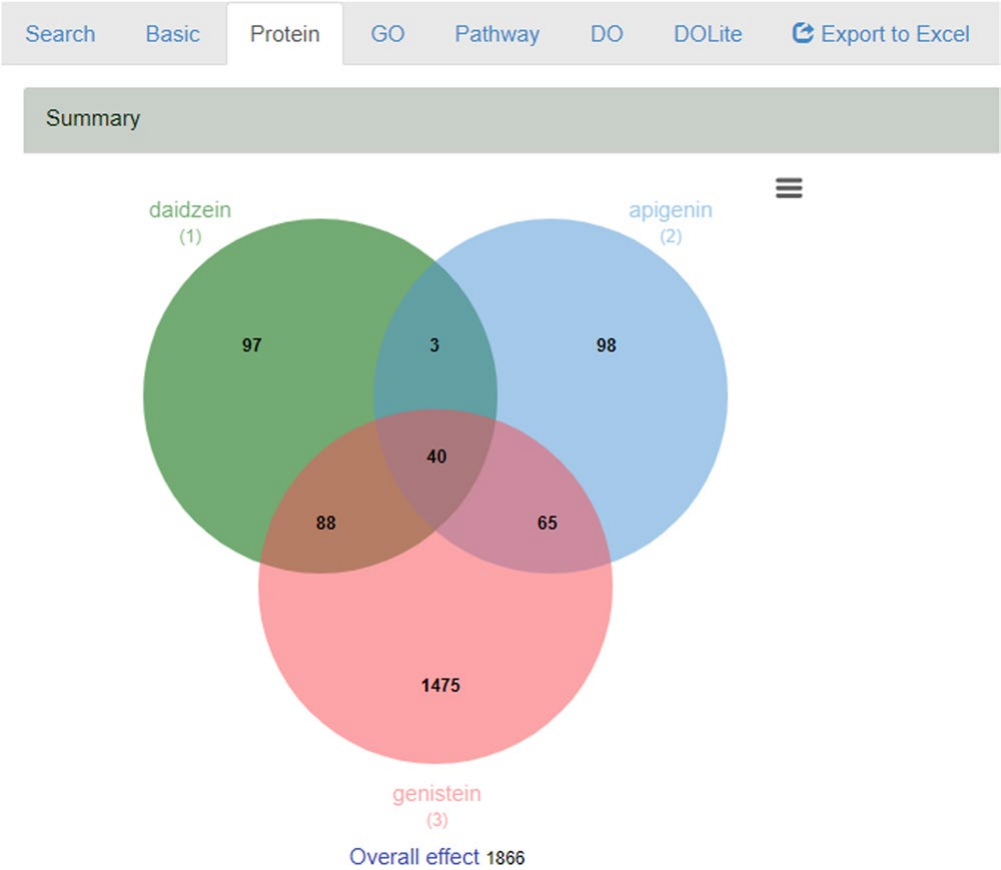
Venn diagram for easy visualization of potential interactions. Except for the basic structure information, Venn diagram analysis is available for protein, GO, pathway, DO and DOLite terms. All numbers in the Venn diagram are clickable for detailed information of associated proteins and *p*-values. The analysis result is downloadable as an Excel file.

For the analysis of shared targets for potential interactions, ChemDIS-Mixture will extract chemical-protein interaction information and present the results as a summarized Venn diagram for easy visualization as shown in Fig. 3. The numbers in the Venn diagram are clickable for acquiring detailed information. For each protein, the Ensemble protein ID, gene symbol, Entrez gene ID, gene name and chemical-protein interaction score provided by STITCH database are browseable. Filter and sort functions have been implemented for each column.

ChemDIS-Mixture offers the analysis functionality of unique and overlapped GO, pathway, DO and DOLite terms among input chemicals. Given multiple chemicals, the enriched GO, pathway, DO and DOLite terms will be first calculated for each chemical based on its interacting proteins. Subsequently, the overlapped and unique terms will be calculated and plotted as a Venn diagram for easy visualization. In addition to the interaction effects, overall effects based on the union of interacting proteins will also be calculated for analyzing the overall effects of a given chemical set. All results are downloadable as an Excel file (Fig. 3).

### Case study: interaction between antituberculosis and antiretroviral drugs

Tuberculosis is one of the most important infections in HIV-infected patients. The drug-drug interaction of combining antituberculosis and antiretroviral therapy has been extensively studied with patient data^20^. Here, we validated the analysis results of ChemDIS-Mixture with the reported concurrent toxicity. Previous studies reported an increased incidence of peripheral neuropathy in patients prescribed isoniazid and stavudine concomitantly^21,22^. In ChemDIS-Mixture, the interaction of isoniazid and stavudine on peripheral neuropathy was identified from the overlapped DO term of peripheral nervous system disease.

Furthermore, the effects of combined use of rifampin and efavirenz compared with efavirenz alone could also be analyzed by using ChemDIS-Mixture. For example, the overlapped DO term of hepatitis (DOID:2237) has been identified for rifampin and efavirenz in this study whose incidence was significantly higher than efavirenz alone (*p* < 0.0001)^21^. However, the incidence of four other diseases including gastrointestinal disturbance, central nervous system disturbance, dermatitis, and peripheral nervous system disease was similar between the two studied groups^21^. ChemDIS-Mixture predicts that dermatitis and peripheral nervous system disease are not potential interaction effects of the combinatory therapy and the prediction is consistent with the previous study^21^. In contrast, two overlapped DO terms of gastrointestinal system disease (DOID:77) and central nervous system disease (DOID:331) were identified by ChemDIS-Mixture. Table 1 shows the adjusted *p*-values for each chemical and the joint *p*-value for the three identified disease terms. From the previous study, the definition of gastrointestinal and central nervous system disturbances is limited to easily observable symptoms such as vomiting and headache^21^. Our analysis suggests that there might be other interaction effects of rifampin and efavirenz on gastrointestinal and central nervous system.

**Table 1:** Selected analysis results of rifampin and efavirenz.

ID	Description	Adj. *p*-value (rifampin)	Adj. *p*-value (efavirenz)	Joint *p*-value
DOID:2237	hepatitis	4.32E-08	0.00906	3.91E-10
DOID:77	gastrointestinal system disease	1.00E-12	0.02271	2.28E-14
DOID:331	central nervous system disease	1.46E-07	0.00394	5.78E-10

A high rate of unexpected hepatotoxicity has been reported in healthy volunteers receiving rifampin and saquinavir/ritonavir^23^. Based on ChemDIS-Mixture hepatotoxicity-related diseases have been successfully identified for the combined use of rifampin and saquinavir/ritonavir. For the combination of rifampin and saquinavir, potential interaction effects on liver-related diseases identified by ChemDIS-Mixture are presented as 5 DO terms of hepatobiliary disease (DOID:3118), hepatitis (DOID:2237), hepatocellular carcinoma (DOID:684), intrahepatic cholestasis (DOID:1852), and hepatic vascular disease (DOID:272). In addition to the abovementioned 5 DO terms, the DO term of hepatoblastoma (DOID:687) was also identified that could be a potential interaction effect of the combination of rifampin and ritonavir. Please note that the potential effects are inferred from the analysis of chemical-protein-disease association and further experiments are required to verify the association. For each association, ChemDIS-Mixture provides target information that may serve as a useful information for generating testable hypotheses.

### Case study: interaction among endocrine disruptors

Dietary flavonoids have a variety of potential effects. However many of them are endocrine disruptors and have been reported to interfere with steroid synthesis. As flavonoids are ubiquitously distributed in foods, human beings may expose to flavonoids mixture via diet. Thus it is important to assess effects of combined exposure to a variety of flavonoids on human health. Soy-based foods contain flavonoids, such as daidzein and genistein^24^. It has been reported that exposure of daidzein, genistein and apigenin mixtures inhibited cortisol, aldosterone and testosterone secretion by human adrenocortical H295R cells in an additive manner, suggesting additive effects of these flavonoids on steroid hormone synthesis^25^. In ChemDIS-Mixture, 40 shared proteins interacted with these three flavonoids were identified as shown in Fig. 3. Among the shared proteins, cytochrome P450 family 19 subfamily A member 1 (CYP19A1), estrogen receptor 1 and 2, and androgen receptor were identified as potential targets responsible for the interaction effect. As CYP19A1 catalyzes many reactions involved in steroidogenesis, these flavonoids could interact with CYP19A1 to disturb steroidogenesis. In addition, the shared targets of estrogen and androgen receptors demonstrate similar endocrine disruption properties of these flavonoids. Please note that flavonoids could have unspecific and low-affinity protein interactions, experiments are required to validate the role of potential targets identified from ChemDIS-Mixture.

Recently, the effects of soy-based foods on human health are controversial. Relief of menopausal symptoms and prevention of heart disease, osteoporosis and cancers are the main health benefits associated with soy foods consumption. On the other hand, intake of soy foods may increase the risk of breast cancer, male hormonal and fertility problems and hypothyroidism^26^. An analysis of daidzein and genistein, the most abundant isoflavones in soy foods, by ChemDIS-Mixture reveals that DO terms of breast cancer (DOID:1612), male breast cancer (DOID:1614), male reproductive system disease (DOID:48), endocrine system disease (DOID:28) and thyroid gland disease (DOID:50) are potential interaction effects of daidzein and genistein. In addition, heart disease (DOID:114) and osteoporosis (DOID:11476) as the beneficial functions of soy foods are also identified. Detailed results are shown in Table 2. Soy consuming populations have been observed to have lower hip fracture rate suggesting the intake of soy-derived isoflavonoid may be effective in maintaining bone health^27^. Several epidemiologic and dietary intervention studies demonstrated the association between phytoestrogens and serum markers of bone turnover, such as bone specific alkaline phosphatase, osteocalcin, insulin-like growth factor I (IGFI), and interleukin 6^28–31^. Two shared proteins of IGFI and tumor necrosis factor superfamily member 11 (commonly known as RANKL) associated with osteoporosis may play roles in the therapeutic effects of soy-derived isoflavones on osteoporosis.

**Table 2:** Selected analysis results of daidzein and genistein.

ID	Description	Adj. *p*-value (daidzein)	Adj. *p*-value (genistein)	Joint *p*-value
DOID:1612	breast cancer	8.63E-16	1.13E-52	9.75E-68
DOID:1614	male breast cancer	0.00959	0.00107	1.00E-05
DOID:48	male reproductive system disease	2.91E-08	0.00001	3.99E-13
DOID:28	endocrine system disease	9.11E-12	5.51E-21	5.02E-32
DOID:50	thyroid gland disease	0.00014	2.27E-14	3.24E-18
DOID:114	heart disease	5.70E-09	1.22E-13	7.00E-22
DOID:11476	osteoporosis	2.84E-10	7.86E-13	2.24E-22

## Discussion

The huge complexity of the assessment of bioactivity and toxicity for mixtures poses the need for novel tools assisting early identification of potential interaction effects. In this study, a novel system ChemDIS-Mixture has been implemented integrating analysis functions in ChemDIS and Venn diagram tools for easy visualization. The functionality of ChemDIS-Mixture has been demonstrated by two case studies. The potential interaction effects of the two case studies were successfully identified. The Venn diagram tool enables quick analysis of overlapped targets, GO, pathway and DO terms. While the analysis of overlapped targets depends on the data of available interacting proteins, poorly characterized chemicals with only a few known chemical-protein interaction data could also benefit from the analysis of enriched GO, pathway and DO terms. Compared with CTD based on only curated chemical-gene interaction data, the utilization of the largest chemical-protein interaction database STITCH integrating many databases enlarges the analysis capability for a large number of chemicals. Please note that the potential interaction effects could be either therapeutic or toxic effects that should be further evaluated by experiments on different conditions of subjects. For example, chemotherapeutic agents are toxic to healthy human beings, but they provide therapeutic effects on cancer patients.

While ChemDIS-Mixture provides useful functions for analyzing potential interaction effects, major limitations are discussed in the follows. First, there is currently no model for estimating dose-response effects of chemical mixtures, which is worth further research. Second, because the inference of interaction effects depends on known interacting proteins, ChemDIS-Mixture is not able to identify all potential effects for chemicals without the complete profile of interacting proteins. Third, although there are a few inhibition and activation data from STITCH, the small number of data limits the application for distinguishing additive, synergistic and antagonistic effects. Future works could be the development and integration of target prediction models that could further enhance the applicability of ChemDIS-Mixture to chemicals without the complete profile of interacting proteins. ChemDIS-Mixture aims to help the early identification of potential endpoints of chemical-chemical interactions whose interaction effects might be further studied by experiments and models such as concentration addition and independent action models. As data grows, ChemDIS-Mixture is expected to be more useful.

## Methods

ChemDIS-Mixture was developed as a subsystem of ChemDIS^11,14,32^ for identifying potential effects and mechanisms of coexposure to multiple chemicals. The core databases integrated in ChemDIS include STITCH for chemical-protein interaction data^16^, Gene Ontology for GO terms representing concepts of molecular functions, cellular components and biological processes^33^, Kyoto Encyclopedia of Genes and Genomes (KEGG)^34^, Reactome^35^ and SMPDB^36^ for pathway information, and Disease Ontology (DO and DOLite) for gene-disease associations^37,38^. STITCH database as the largest chemical-protein interaction database aggregating multiple databases such as CTD^12^, ChEMBL^17^, DrugBank^39^, KEGG^34^ and Reactome^35^ largely increase the coverage of diverse chemicals in ChemDIS-Mixture. The core databases provide essential data on interacting genes for a given chemical that will be further connected to the GO, pathway and DO terms. The user interface and computation modules were implemented using PHP, JavaScript and GO languages. Venn diagrams were dynamically generated using jvenn^40^ for the visualization of overlapped genes/pathways/diseases. For the prioritization of potential interaction effects, a generalized form of joint *p*-value ${p}_{j}=\prod _{i=1}^{n}{p}_{i}$ will be calculated, where *p*_*i*_ represents the adjusted *p*-value for chemical *i*. The joint *p*-value represents the overall significance of a given effect affected by multiple chemicals that has been shown to be effective for the identification of enriched terms supported by multiple datasets^41^.
